# Reassessing the Role of Regular Physical Examination in Post-treatment Breast Cancer Surveillance

**DOI:** 10.1245/s10434-026-19227-7

**Published:** 2026-02-12

**Authors:** Anna Levine, Luke Terrian, Daniel Bishay, Kelsey Lawrence, G. Paul Wright, Jessica Thompson

**Affiliations:** 1 Division of Cancer Health, Corewell Health West, Grand Rapids, MI USA; 2 Michigan State University College of Human Medicine, Grand Rapids, MI USA

**Keywords:** Breast cancer, Physical examination, Survivorship, Cancer screening, Surveillance, Recurrence detection, Oncology practice

## Abstract

**Background:**

Routine provider-performed physical examinations remain a standard element of breast cancer survivorship despite increasing evidence that diagnostic yield is limited. With growing survivorship populations and mounting demands on oncology services, reassessment of the value of this practice is warranted. This study sought to evaluate the diagnostic yield and prognostic relevance of surveillance physical exams.

**Methods:**

A retrospective review was conducted of women with breast cancer treated between January 2019 and December 2023 at a single tertiary center. Surgical oncology survivorship visits >90 days postoperatively were analyzed. Recurrence events were categorized by initial detection modality: provider-performed physical examination, patient-reported, screening imaging, or symptom-prompted non-breast imaging.

**Results:**

Among 3337 patients, 131 recurrences (3.9%) occurred after a median of 20 months. Patients collectively attended 12,840 survivorship visits; only eight recurrences (6.1% of all recurrences; 0.06% of examinations) were first detected by physical examination. By comparison, 32 (24.4%) were patient-reported, 38 (29.0%) were detected on screening imaging, and 53 (40.5%) were identified by non-breast diagnostic imaging. When stratified by detection method, exam-detected recurrences were evenly divided between locoregional and combined locoregional/distant disease, whereas patient-reported and screening-detected recurrences were predominantly locoregional, at 72% and 92%, respectively.

**Conclusions:**

Breast and chest wall examinations accounted for a small proportion of recurrence detections despite thousands of survivorship visits annually. Most clinically meaningful recurrences were identified by imaging or patient self-report. These findings support survivorship strategies that prioritize imaging adherence and structured symptom assessment, with less emphasis on routine physical examination as a primary detection modality.

Post-treatment surveillance after curative treatment for breast cancer aims to identify recurrence or new primary disease at an early and treatable stage. Current guidelines endorse regularly scheduled in-person survivorship visits incorporating clinical examinations every 3 to 12 months.^1–3^ These recommendations reflect the premise of supporting timely recognition of recurrent disease. While this approach has been long-established, the relative contribution of physical examinations to relapse detection in present-day practice is a subject of debate, especially in the context of widespread use of breast imaging and increased patient awareness.^4–8^

With breast cancer incidence rising and survival steadily improving, there is a growing population of long-term survivors requiring ongoing follow-up.^9,10^ This trend places considerable demands on healthcare systems already challenged by resource constraints, workforce limitations, and the need for cost containment.^11,12^ Current surveillance guidelines apply broadly to all patients, yet the actual benefit of standardized strategies, particularly the incremental value of physical exams in detecting clinically significant relapses, remains unclear. These considerations highlight the need to reassess whether generalized approaches to surveillance adequately address the heterogeneity of breast cancer survivors.

To address this gap, we conducted a retrospective cohort study to evaluate the yield of routine provider-performed physical examinations in identifying recurrent breast cancer in patients previously treated for in situ or invasive disease. Understanding the relative contribution of physical exams in contemporary surveillance practice can inform follow-up strategies that optimize resource allocation, reduce unnecessary patient burden, and ensure timely recognition of clinically meaningful events.

## Patients and Methods

### Patient Selection

We conducted a single-institution retrospective cohort study at Corewell Health West, MI, USA, to evaluate the role of routine provider-performed physical breast and/or chest wall examinations in the survivorship setting. Patients were identified through a query of the institutional cancer registry, which is prospectively maintained. Eligible patients were assigned female sex at birth, aged ≥18 years, with a stage 0–III breast cancer diagnosis, who underwent either partial or total mastectomy at Corewell Health West between January 1, 2018, and December 31, 2023. Documentation of at least one physical breast and/or chest wall examination in the breast surgical oncology clinic after the 90-day postoperative period following the index operation was required for inclusion.

The analysis was restricted to surgical oncology survivorship visits to ensure consistency and completeness of clinical documentation. At our institution, a substantial proportion of patients receive medical oncology or primary care follow-up outside the Corewell Health system, for which documentation is not uniformly available in the electronic medical record (EMR). In addition, breast and chest wall examinations within the surgical oncology practice follow a standardized approach, allowing for more uniform assessment of provider-performed examinations. Limiting the analysis to surgical oncology visits minimized bias related to incomplete follow-up capture while ensuring consistency in the exposure of interest. The 90-day postoperative threshold was selected to exclude postoperative encounters, as well as disease detected shortly after surgery that is more likely to represent persistent or previously unrecognized disease rather than true biologic recurrence. This approach allowed the analysis to focus on survivorship-phase surveillance.

Patients were excluded if surgery was completed at an outside institution, if lost to follow-up after the 90-day postoperative period, or if diagnosed with de novo metastatic (stage IV) disease within 4 months of initial diagnosis, in accordance with National Comprehensive Cancer Network staging definitions. Patients with primary or secondary breast sarcomas were excluded because of distinct biology, surveillance approaches, and recurrence patterns. Patients assigned male sex at birth were also excluded because of differences in tumor biology, treatment paradigms, and survivorship surveillance. The institutional review board approved this study with a waiver of informed consent because of the retrospective nature of the analysis.

### Data Collection

Eligible patients were first identified through registry diagnosis codes, after which detailed chart review was performed. Demographic characteristics were abstracted from the EMR. Tumor-specific data were obtained from both the registry and the EMR and included age at diagnosis, histologic subtype, T category, nodal status, grade, estrogen receptor (ER) status, progesterone receptor (PR) status, and human epidermal growth factor receptor 2 (HER2) expression. Operative details (surgical date, type of breast and axillary surgery) were recorded in addition to information regarding receipt of chemotherapy, endocrine therapy, targeted therapy, immunotherapy, and/or radiation. Information regarding the clinical presentation at initial breast cancer diagnosis was not systematically collected and therefore not analyzed in relation to recurrence detection patterns.

Recurrent events were identified from the EMR. New primary breast cancers were not included as recurrence events. For each relapse, reviewers recorded the anatomic site of disease (local, regional, and/or distant) and the initial mode of detection. Method of detection was classified into four categories: (1) provider-initiated by surgical oncology, medical oncology, plastic surgery, or primary care physical examination; (2) patient-reported changes, subsequently correlated with exam and imaging; (3) screening imaging–detected through mammography, breast magnetic resonance imaging (MRI), or ultrasound; or (4) non-breast diagnostic imaging prompted by new non-breast clinical complaints. Physical examination-detected recurrences were defined as those in which a new abnormal physical finding (e.g., mass, nodularity, skin change, nipple change) identified by a healthcare provider during an in-person examination directly prompted further diagnostic evaluation. Symptoms elicited by history alone without a corresponding abnormal physical exam finding were not included in this category. Patient-reported recurrences were defined as those initiated by patient-identified symptoms or changes elicited during history taking at a survivorship visit or reported outside of a scheduled surveillance encounter (e.g., phone calls, patient portal messages, unscheduled visits) that subsequently prompted diagnostic workup. Screening imaging-detected recurrences were defined as asymptomatic findings identified on routine surveillance imaging performed according to guideline-based follow-up. Mammograms performed in asymptomatic patients as part of routine surveillance were classified as screen-detected regardless of whether they were ordered as “diagnostic” because of the proximity to treatment. Non-breast diagnostic imaging-detected recurrences were defined as those prompted by new symptoms in the absence of an abnormal physical examination, which led to diagnostic imaging. These symptoms were identified either out of routine oncology follow-up (e.g., emergency department or urgent care encounters, or non-oncology outpatient evaluations) or during review of systems at a scheduled oncology visit that subsequently prompted diagnostic imaging. This category primarily included computed tomography (CT), positron emission tomography (PET)/CT, bone scans, and MRI of non-breast sites. Recurrences in this category were not classified as examination-detected unless a corresponding abnormal physical exam finding directly initiated further diagnostic workup. Survival status was collected from the EMR, and cause of death was determined through individual chart review when applicable and available.

The occurrence of survivorship clinic encounters and breast imaging studies (mammogram, ultrasound, MRI) obtained beyond the 90-day postoperative period was recorded. At our institution, screening imaging and survivorship visits are scheduled independently, with the 6-month survivorship visits typically preceding routine surveillance imaging. As a result, provider-performed exams are not routinely informed by contemporaneous breast imaging results, enabling evaluation of their diagnostic yield as an independent detection modality in our study analysis. Digital breast tomosynthesis was the standard modality for annual screening mammography. For selected patients, alternating mammography and breast MRI at 6-month intervals was recommended based on individual risk and treatment history. Molecular breast imaging and contrast-enhanced mammography were not available during the study period. Breast ultrasound was performed for diagnostic indications at the discretion of the treating provider and radiologists.

### Statistical Analysis

The primary study endpoint was the proportion of breast cancer recurrences initially identified on provider-performed physical examination compared with other detection methods (surveillance imaging, patient self-detection, or alternative non-breast imaging). Descriptive statistics were used to summarize baseline characteristics, treatment variables, and outcomes. Categorical variables were presented as counts and percentages and compared between patients with a documented recurrence (*n* = 131) and those without (*n* = 3206) using Fisher’s exact test or the *χ*^2^ test, as appropriate. Continuous variables were summarized as means with standard deviations or medians with interquartile range (IQR), depending on data distribution assessed by the Shapiro–Wilk test, and compared using the independent t-test or the Mann–Whitney U test, as appropriate. All tests were two-sided, and statistical significance was defined as *p* <0.05. Analyses were performed using R, version 1.1.442 (R Foundation for Statistical Computing, Vienna, Austria).

## Results

### Patient Demographics

The study cohort comprised 3337 women, representing 3453 total breast cancer diagnoses, including 116 patients with synchronous bilateral disease. Median age at diagnosis was 62 years (IQR 52–70). Descriptive characteristics of the study population are summarized in Table 1. During the 5-year study period, patients collectively attended 12,840 surgery-specific survivorship visits, excluding postoperative visits within 90 days of index surgery. The median number of visits was three per patient (IQR 2–5). Median follow-up for the overall cohort, as well as for patients without recurrence, was 18 months (IQR 12–36).

**Table 1:** Demographic and clinicopathologic features of study population at initial diagnosis of stage 0–III breast cancer (2019–2023), by subsequent recurrence status

Variable	No breast cancer recurrence (*n* = 3206)	Breast cancer recurrence (*n* = 131)	*p-*value
Age at diagnosis, years	62 (52.0–70.0)	63 (50.5–74.0)	0.59
Race			0.67
White	3022 (94.3)	123 (93.9)	
Black	104 (3.2)	6 (4.6)	
Asian	43 (1.3)	2 (1.5)	
Unreported	37 (1.2)	0 (0)	
Ethnicity			0.70
Non-Hispanic	3116 (97.2)	128 (97.7)	
Hispanic	74 (2.3)	3 (2.3)	
Unreported	16 (0.5)	0 (0)	
Insurance			0.29
Private	1540 (48)	55 (42)	
Medicare	1399 (43.6)	61 (46.6)	
Medicaid	215 (6.7)	13 (9.9)	
Tricare	16 (0.5)	0 (0)	
Other	29 (0.9)	1 (0.8)	
Uninsured	7 (0.2)	1 (0.8)	
Menopausal status			0.85
Premenopausal	660 (20.6)	30 (22.9)	
Perimenopausal	60 (1.9)	2 (1.5)	
Postmenopausal	2480 (77.3)	99 (75.6)	
Unverified	6 (0.2)	0 (0)	
BMI, kg/m^2^	28.1 ± 6.4	28.6 ± 7.1	0.17
Histology			0.002
DCIS	580 (18.1)	15 (11.5)	
IDC	1743 (54.4)	80 (61.1)	
ILC	358 (11.2)	12 (9.2)	
MIDLC	404 (12.6)	16 (12.2)	
Other	121 (3.8)	8 (6.1)	
Clinical T category			<0.001
Tis	624 (19.5)	14 (10.7)	
T1mi	25 (0.8)	0 (0)	
T1	1767 (55.1)	46 (35.1)	
T2	558 (17.4)	48 (36.6)	
T3/T4 excluding IBC	118 (3.7)	14 (10.7)	
IBC	7 (0.2)	7 (5.3)	
Occult	7 (0.2)	0 (0)	
Paget’s	6 (0.2)	0 (0)	
Incidental	94 (2.9)	2 (1.5)	
Nodal status			<0.001
N0	2489 (77.6)	81 (61.8)	
N1	601 (18.7)	35 (26.7)	
N2/N3	116 (3.6)	15 (11)	
ER status			0.001
Positive	2982 (93)	110 (84)	
Negative	224 (7)	21 (16)	
PR status			0.002
Positive	2589 (81)	95 (72.5)	
Negative	608 (19)	36 (27.5)	
HER2 neu status			0.09
Positive	403 (12.6)	21 (16)	
Negative	2803 (87.4)	110 (84)	
Neoadjuvant systemic therapy administered	387 (12.1)	38 (29)	< 0.001
Index surgery			< 0.001
Breast conserving	2229 (69.5)	75 (57.3)	
Mastectomy	934 (29.1)	30 (22.9)	
MRM	40 (1.2)	26 (19.8)	
ALND only	3 (0.1)	0 (0)	

Data are presented as median (interquartile range), mean ± standard deviation, or *n* (%) unless otherwise indicated

ALND, axillary lymph node dissection; BMI, body mass index; DCIS, ductal carcinoma in situ; ER, estrogen receptor; HER2, human epidermal growth factor receptor 2; IBC, inflammatory breast cancer; IDC, invasive ductal carcinoma; ILC, invasive lobular carcinoma; MIDLC, mixed invasive ductal and lobular carcinoma; MRM, modified radical mastectomy; PR, progesterone receptor

Statistically significant differences between patients with and without recurrence were observed across multiple clinicopathologic characteristics (Table 1). Patients with recurrence were more likely to have invasive disease (88.5% vs 81.9%), higher clinical T category (T2–T4, 52.6% vs 21.3%), and nodal involvement (node positive, 37.7% vs 22.3%) than were those without recurrence. Tumors in patients with recurrence were more frequently ER negative (16% vs 7%) and PR negative (27.5% vs 19%), and these patients were more likely to have received neoadjuvant systemic therapy (29% vs 12.1%). No significant differences were observed by race, ethnicity, insurance status, menopausal status, body mass index, or age at diagnosis.

### Yield of Provider-Performed Physical Examination

A total of 131 recurrences (3.9%) were identified during the study period. Baseline characteristics of patients diagnosed with recurrence are presented in Table 2. Recurrences were classified based on the initial clinical indication for imaging rather than the setting in which the imaging was obtained. These patients collectively attended 418 surgery-specific survivorship visits between index surgery and relapse, with a median of two visits per patient (IQR 1–4).

**Table 2:** Index tumor characteristics and initial therapies rendered among patients later diagnosed with recurrent breast cancer, categorized by mode of recurrence detection

Characteristic	Physical exam (*N*=8)	Patient reported (*N*=32)	Screening imaging (*N*=38)	Non-breast imaging (*N*=53)	*P-*Value
Age at diagnosis, years	75 (56.5–81.5)	52 (41.8–77.3)	70 (62.0–77.5)	62 (51.0–67.0)	0.017
Race					0.74
White	7 (87.5)	29 (90.6)	36 (94.7)	51 (96.2)	
Black	1 (12.5)	2 (6.2)	2 (5.3)	1 (1.9)	
Asian	0 (0)	1 (3.1)	0 (0)	1 (1.9)	
Insurance					0.18
Private	2 (25)	16 (50)	15 (39.5)	22 (41.5)	
Medicare	5 (62.5)	12 (37.5)	23 (60.5)	21 (39.6)	
Medicaid	1 (12.5)	4 (12.5)	0 (0)	8 (15.1)	
Other	0 (0)	0 (0)	0 (0)	1 (1.9)	
Uninsured	0 (0)	0 (0)	0 (0)	1 (1.9)	
Pathogenic mutation					
BRCA1	1	0	0	2	
BRCA2	0	2	0	1	
ATM	1	2	0	0	
CHEK2	0	0	3	1	
PALB2	0	0	0	1	
RAD51C/D	0	0	0	1	
Synchronous bilateral cancer	0 (0)	1 (3.1)	2 (5.3)	4 (7.5)	0.26
Histology					0.041
DCIS	1 (12.5)	2 (6.2)	12 (31.6)	0 (0)	
IDC	4 (50)	25 (78.1)	18 (47.4)	33 (62.3)	
ILC	1 (12.5)	0 (0)	2 (5.3)	9 (17)	
MIDLC	2 (25)	2 (6.2)	5 (13.2)	7 (13.2)	
Micropapillary	0 (0)	1 (3.1)	0 (0)	0 (0)	
Mucinous	0 (0)	0 (0)	1 (2.6)	1 (1.9)	
Metaplastic	0 (0)	0 (0)	0 (0)	1 (1.9)	
Other	0 (0)	2 (6.2)	0 (0)	2 (3.8)	
Tumor grade (invasive)					0.047
Low	2 (25)	5 (15.6)	12 (31.6)	6 (11.3)	
Intermediate	4 (50)	11 (34.4)	18 (47.4)	18 (34)	
High	2 (25)	16 (50)	6 (15.8)	27 (50.9)	
Unknown	0 (0)	0 (0)	2 (5.3)	2 (3.8)	
Clinical T category					0.003
DCIS	1 (12.5)	2 (6.2)	11 (28.9)	0 (0)	
T1	3 (37.5)	17 (53.1)	15 (39.5)	11 (20.8)	
T2	1 (12.5)	10 (31.2)	10 (26.3)	27 (50.9)	
T3	2 (25)	1 (3.1)	0 (0)	8 (15.1)	
T4	1 (12.5)	0 (0)	0 (0)	2 (3.8)	
Inflammatory	0 (0)	2 (6.2)	0 (0)	5 (9.4)	
Unknown	0 (0)	0 (0)	2 (5.3)	0 (0)	
Clinical N category					<0.001
N0	6 (75)	25 (78.1)	34 (89.5)	26 (49.1)	
N1	2 (25)	6 (18.8)	1 (2.6)	20 (37.7)	
N2	0 (0)	0 (0)	1 (2.6)	4 (7.5)	
N3	0 (0)	1 (3.1)	0 (0)	3 (5.7)	
Unknown	0 (0)	0 (0)	2 (5.3)	0 (0)	
Clinical stage					<0.001
DCIS	1 (12.5)	2 (6.2)	11 (28.9)	0 (0)	
I	4 (50)	18 (56.3)	20 (52.6)	14 (26.4)	
II	0 (0)	7 (21.9)	4 (10.5)	24 (45.3)	
III	3 (37.5)	5 (15.6)	1 (2.6)	15 (28.3)	
Unknown	0 (0)	0 (0)	2 (5.3)	0 (0)	
Receptor subtype					0.002
HR+ DCIS	1 (12.5)	1 (3.1)	7 (18.4)	0 (0)	
HR– DCIS	0 (0)	1 (3.1)	5 (13.2)	0 (0)	
HR+ HER–	4 (50)	19 (59.4)	20 (52.6)	28 (52.8)	
HR+ HER+	0 (0)	0 (0)	3 (7.9)	2 (3.8)	
HR– HER+	0 (0)	2 (6.2)	0 (0)	4 (7.5)	
HR– HER–	3 (37.5)	9 (28.1)	3 (7.9)	19 (35.8)	
Received neoadjuvant systemic therapy	2 (25)	8 (25)	4 (10.6)	24 (45.3)	0.003
Index surgery					<0.001
Breast conserving	3 (37.5)	20 (62.5)	31 (81.6)	21 (39.6)	
Mastectomy	2 (25)	8 (25)	6 (15.8)	13 (24)	
MRM	3 (37.5)	4 (12.5)	1 (2.6)	19 (35.8)	
Axillary surgery					<0.001
None	3 (37.5)	5 (15.6)	18 (47.4)	2 (3.8)	
SLNB	2 (25)	20 (62.5)	17 (44.7)	27 (50.9)	
ALND	3 (37.5)	7 (21.9)	3 (7.9)	24 (45.3)	
Reconstruction performed	1 (12.5)	7 (21.9)	3 (7.9)	7 (13.2)	0.87
Received adjuvant chemotherapy	2 (25)	8 (25)	5 (13.2)	18 (34)	0.23
Received adjuvant radiation	2 (25)	9 (28.1)	7 (18.4)	38 (71.7)	<0.001
Received adjuvant endocrine therapy	2 (25)	11 (34.4)	11 (28.9)	20 (37.7)	0.52
Received targeted anti-HER2 therapy	0 (0)	2 (6.2)	3 (7.9)	7 (13.2)	0.52
Received immunotherapy	0 (0)	1 (3.1)	0 (0)	4 (7.5)	0.09
Received CDK4/6 inhibitor	0 (0)	0 (0)	1 (2.6)	2 (3.8)	0.27
Number of surgery-specific survivorship visits before recurrence	2 (2.0–3.5)	2.5 (1.0–5.0)	3 (2.0–4.75)	2 (1.0–4.0)	0.61

Data are presented as median (interquartile quartile), mean ± standard deviation, or *n* (%) unless otherwise indicated

ALND, axillary lymph node dissection; DCIS, ductal carcinoma in situ; HER, human epidermal growth factor receptor; HR, hormone receptor; IDC, invasive ductal carcinoma; ILC, invasive lobular carcinoma; MIDLC, mixed invasive ductal and lobular carcinoma; MRM, modified radical mastectomy; SLNB, sentinel lymph node biopsy

Of the 12,840 survivorship visits during the study period, only eight (0.06%) provider-performed breast or chest wall examinations led to the initial detection of a recurrence. Among the eight recurrences initially detected by physical examination, all were first identified during clinical encounters outside of scheduled surgical oncology survivorship visits. Two were identified by plastic surgery during tissue expander-implant exchange, one by a primary care provider during an annual physical examination, and five by medical oncology during routine surveillance follow-up. In all cases, these findings prompted referral to surgical oncology for confirmatory evaluation and management. Encounters with non-surgical oncology specialties were not included as surveillance visits in the primary analysis, consistent with the predefined study design.

The overall yield of physical examination as the index detection modality was 2.4 per 1000 patients. By comparison, 32 recurrences were patient-reported, 38 were detected through screening imaging, and 53 were identified on other non-breast diagnostic imaging prompted by new clinical complaints. Among recurrences detected through other non-breast diagnostic imaging, the majority were prompted by new symptoms in the absence of abnormal physical exam findings. A subset of these events was initiated by symptoms elicited during review of systems (e.g., pulmonary, neurologic, musculoskeletal, gastrointestinal) at scheduled oncology visits, which subsequently prompted diagnostic imaging. The remaining recurrences in this category occurred outside of routine survivorship follow-up and were identified on cross-sectional imaging obtained during external evaluations performed for workup of new or progressive symptoms that led to the diagnosis of recurrent disease.

### Characteristics of Recurrent Cancers

Among the 131 recurrences, 64 (49%) were locoregional, 56 (43%) were distant, and 11 (8%) were both locoregional and distant. Median age at initial diagnosis was 63 years (IQR 50.5–74), and the median time to recurrence was 20 months (IQR 12–30.5). When stratified by detection modality, as demonstrated in Table 3, the physical exam cohort (*n*=8) included four patients with locoregional recurrence and four with combined locoregional and distant recurrence. Patient-reported recurrences (*n*=32) were primarily locoregional (72%), with 16% combined and 12% distant. Screening-detected recurrences (*n*=38) were predominantly locoregional (92%), and those identified by non-breast imaging (*n*=53) were overwhelmingly distant (94%). Additional individual-level characteristics, treatments, detection settings, and outcomes for the eight recurrences detected by provider-performed examinations are summarized in Table 4.

**Table 3:** Characteristics and outcomes of breast cancer recurrence cases, stratified by recurrence detection modality

Characteristic	Physical exam (*N*=8)	Patient reported (*N*=32)	Screening imaging (*N*=38)	Non-breast imaging (*N*=53)
*Recurrence type*				
LRR	4 (50)	23 (71.9)	35 (92.1)	2 (3.8)
LRR/distant	4 (50)	4 (12.5)	2 (5.3)	1 (1.8)
Distant	0 (0)	5 (15.6)	1 (2.6)	50 (94.3)
Time to recurrence, months	16.5 (14.5–22.5)	20 (11.0–30.0)	24 (12.5–34.5)	19 (12.0–27.0)
Deaths during study period	5 (50)	10 (31.2)	3 (7.9)	26 (49.1)
Breast-cancer related deaths	4	8	0	25
Survival after recurrence, months	16 (12.0–17.0)	24.5 (21.5–30.0)	–	13 (2.5–27.0)

Data are presented as median (interquartile range) or *n* (%) unless otherwise indicated. Formal statistical comparisons between detection modality cohorts were not performed because of the small subgroup sizes and limited event counts

LRR, locoregional recurrence

**Table 4:** Clinicopathologic features and outcomes of patients with breast cancer recurrence detected by provider-performed breast or chest wall examination

Pt	Age at diagnosis, years	Race/ethnicity	Genetic testing	Initial diagnosis and stage	Primary treatment	Relapse detection	Relapse type	Time to relapse	Outcome
1	30	Other Hispanic	BRCA1	MIDLC, TNBC, grade 2 cT3N1 stage III	NAC; MRM, ypT3N2; adjuvant capecitabine	Medical oncology	Local distant (bone)	15 mo	Died 16 mo after relapse ^a^
2	83	White, non-Hispanic	Not performed	IDC, TNBC, grade 2, cT3N1 stage III	Upfront MRM, pT3N2; adjuvant chemotherapy; declined RT	Medical oncology	Local distant (bone)	13 mo	Died 3 mo after relapse^b^
3	81	White, non-Hispanic	Not performed	IDC, HR+ HER2-, grade 2, cT1N0, stage I	Upfront BCS, pT1Nx declined RT; accepted ET	Medical oncology	Local	16 mo	Alive at 88 y
4	55	White, non-Hispanic	ATM	MIDLC, HR+ HER2-, grade 1, cT4N0, stage III	NST^c^; BLM/SLNB, ypT1N0; declined RT accepted ET	Medical oncology	Local	31 mo	Alive at 62 y
5	79	White, non-Hispanic	Negative	IDC, HR+ HER2-, grade 3, cT1N0, stage I	Upfront BCS, pT2Nx; adjuvant RT; adjuvant ET	Medical oncology	Loco-regional	22 mo	Alive at 88 y
6	87	White, non-Hispanic	Negative	IDC, TNBC, grade 3, cT1N0, stage I	Upfront BCS, pT1Nx; adjuvant RT	Primary care	Local distant (lung)	24 mo	Died 15mo after relapse^a^
7	71	White, non-Hispanic	Not performed	ILC, HR+ HER2-, grade 1, cT2N0, stage I	Upfront MRM, pT3N2; Declined all adjuvant therapy	Plastic surgery	Local distant	12 mo	Died 15 mo after relapse^b^
8	57	Black, non-Hispanic	Negative	DCIS, HR+, grade 2	BLM/SLNB	Plastic surgery	Local (DCIS)	17 mo	Alive at 60 y

BCS, breast conserving surgery; BLM, bilateral mastectomy; DCIS, ductal carcinoma in situ; ET, endocrine therapy; HER, human epidermal growth factor receptor; HR, hormone receptor; IDC, invasive ductal carcinoma; ILC, invasive lobular carcinoma; MIDLC, invasive mixed ductal and lobular carcinoma; mo, months; MRM, modified radical mastectomy; NAC, neoadjuvant chemotherapy; NST, neoadjuvant systemic therapy; pt, patient; RT, radiation therapy; SLNB, sentinel lymph node biopsy; TNBC, triple negative breast cancer; y, years

^a^Received palliative systemic therapy

^b^Entered hospice without receipt of systemic therapy

^c^Neoadjuvant systemic therapy consisted of anastrozole and chemotherapy

Although patient-specific education data were not available, insurance status and race were examined as surrogate markers of socioeconomic factors. Black patients and non-private insurance represented a higher proportion of exam-detected recurrences than other detection modalities. Although these differences were not statistically significant, it raises the possibility of differential access to or utilization of surveillance imaging and symptom-based reporting.

### Mortality

During the study period, 37 breast cancer-related deaths occurred. Outcomes varied by detection modality. The four patient deaths in the provider-performed physical exam cohort were all attributable to breast cancer. In the patient-reported group, 10 deaths occurred, eight of which were breast cancer-related. Among patients with screening-detected recurrences, three deaths occurred, none attributable to breast cancer. In the non-breast imaging cohort, 26 deaths occurred, 25 of which were breast cancer-related. Median survival following recurrence diagnosis also varied by detection method: 16.5 months in the physical exam group, 24.5 months in the patient-reported group, not applicable in the screening cohort (no breast cancer-related deaths), and 13 months in the non-breast imaging group. Formal statistical comparison between cohorts was not performed because of the small subgroup sizes and limited event counts.

## Discussion

Survivorship follow-up remains a central component of breast cancer care, yet the specific contribution of routine provider-performed breast and chest wall examinations to recurrence detection is incompletely defined. Of the 131 recurrences (3.9%) in this study cohort of 3337 patients, only eight were initially identified through abnormal physical examination findings. In contrast, the majority of recurrences were discovered through surveillance imaging or symptom-driven diagnostic evaluation. These findings suggest that, in contemporary practice, the incremental diagnostic value of routine physical examination is limited.

It is important to distinguish the limited diagnostic yield of provider-performed breast and chest wall examinations from the broader clinical value of survivorship visits. Although abnormal physical exam findings rarely served as the initial basis for recurrence detection, many relapses in this study, particularly distant events, were identified through symptom assessment rather than examination. Symptom-prompted imaging arose both during review of systems at scheduled oncology visits and during evaluations outside routine follow-up. Collectively, these observations underscore that history taking, symptom recognition, and timely diagnostic evaluation account for most clinically meaningful detections, rather than physical exam alone.

Recent data have highlighted the evolving landscape of breast cancer surveillance. Our findings align with prior reports and systematic reviews that have reinforced the modest contribution of physical exams when compared with high-quality radiologic studies and active patient engagement.^6,7,13,14^ Beltran-Bless et al.^6^ observed that the majority of local recurrences were identified by patients (54.7%) or routine mammography (41.5%) with only a marginal proportion (≤2%) identified during scheduled provider examinations. Their updated analysis reinforced this pattern in a larger cohort, with 5 of 390 relapses (1.3%) detected by healthcare providers during routine follow-up encounters, only one of which was potentially curable.^7^ These findings are further supported by systematic reviews that have assessed the relative performance of surveillance strategies. In their 2007 systematic review, Montgomery et al.^4^ found that nearly 40% of local recurrences were detected by mammography, and a similar percentage (41%) were discovered by patients. More recently, Godding et al.^8^ confirmed that physical exams play a secondary role, with most recurrences detected either by self-report or imaging. In this study, recurrences initially detected by physical examination were identified during encounters with other specialists and subsequently referred for surgical oncology evaluation. This pattern suggests potential redundancy in routine physical examinations across specialties and warrants further evaluation of whether surgical oncology-specific surveillance examinations could be selectively de-escalated in appropriately followed patients.

Across contemporary series, the majority of relapses after treatment for early-stage breast cancer are distant rather than locoregional, and therefore infrequently amenable to curative intervention.^6,7,15–17^ In our cohort, the prognostic implications of detection modality were notable. Screen-detected recurrences were almost exclusively locoregional and associated with no breast cancer-related deaths during the study period, whereas patient-reported events were frequently locoregional and associated with more favorable survival than exam-detected events. In contrast, recurrences identified through non-breast diagnostic imaging were overwhelmingly distant and carried the poorest prognosis. These differences underscore that the value of a detection modality depends not only on frequency of detection but also its capacity to identify disease at a stage amenable to intervention. Although index surgery differed significantly across recurrence cohorts, exam-detected events were not concentrated among patients who underwent mastectomy, suggesting that surgical approach alone does not identify a subgroup in whom routine physical examination provides greater diagnostic yield.

Socioeconomic and racial factors may influence surveillance pathways and detection patterns. Although individual-level education and socioeconomic status was not available in this retrospective analysis, insurance status was examined as a surrogate marker of access to care; however, interpretation was limited by the small subgroup sizes. Although the proportion of patients with private insurance appeared lower in the physical exam-detected cohort, formal statistical testing did not demonstrate a significant association between insurance status and detection modality. Notably, differences by race were observed, with Black patients representing a higher proportion of physical exam-detected recurrences compared with other detection modalities. This observation raises the possibility that additional unmeasured factors, such as health literacy, access to imaging, or symptom reporting, may influence how and when relapses are identified. These findings underscore the importance of ensuring that evolving survivorship models do not inadvertently exacerbate existing disparities.

From a systems perspective, routine survivorship care is resource intensive, requiring thousands of encounters annually across multidisciplinary teams, yet the proportion of recurrences detected through provider-performed breast and chest wall examination is exceedingly small. When considered alongside the expanding population of breast cancer survivors amid ongoing workforce shortages and increasing pressure on oncology services, the incremental diagnostic contribution of routine physical exam appears limited when considered independently from other components of survivorship care.^11,18,19^ A recent analysis estimated that nearly 88,000 follow-up visits would be necessary to detect a single potentially curable recurrence by physical exam alone, emphasizing the need to recalibrate survivorship paradigms.^7^ Future strategies may be better oriented around adherence to high-quality imaging, structured patient education regarding symptoms of relapse, and incorporation of flexible care delivery models such as telehealth. Structured tools, such as symptom checklists, electronic reminders, or nurse-led telephonic follow-up, could support early reporting and reduce reliance on visit-based physical examinations.^19–21^

Alternative models of survivorship care have demonstrated promise in international settings. Risk-adapted pathways incorporating patient-initiated follow-up, telehealth, and nurse-led surveillance have reduced routine visit volume without compromising recurrence outcomes or patient satisfaction.^19,22,23]^ Early evaluations suggest that alternating telehealth and in-person encounters or replacing routine appointments with on-demand access can decrease costs and administrative burden. Translation of these approaches to the United States will require thoughtful implementation, including structured triage systems, patient education, and coordination across oncology and primary care. Importantly, such models should be designed to complement, not replace, clinical oversight and ensure access for patients with higher-risk disease or greater barriers to care. Although the diagnostic yield of routine physical examination appears limited, survivorship encounters allow providers to address functional recovery, assess treatment-related morbidity, and reinforce lifestyle modifications and preventive health measures. Survivorship appointments also serve as a forum to support psychosocial well-being, body image concerns, and long-term treatment effects that may impact quality of life. Many of these elements of care could be effectively maintained through alternative formats, enabling continuity of comprehensive survivorship support while reducing reliance on in-person visits.

This study has a number of limitations. As a single-institution retrospective review, generalizability may be constrained. Education and socioeconomic variables were not available, limiting assessment of their impact on detection pathways. Although recurrence events were identified within our health system, events managed outside the institution may have been missed. The study was designed to evaluate the diagnostic yield of provider-performed breast and chest wall exams for confirmed recurrences but did not capture other outcomes of survivorship care, such as adherence to screening imaging or adjuvant therapies. Additionally, data on workup for findings that did not ultimately represent recurrence were not collected, which limits our ability to quantify false-positive examinations or associated follow-up testing. Lastly, our institutional model involves independent scheduling of radiology and clinic encounters, which may not reflect practices at other centers and could impact attribution of the initial detection modality. Despite these limitations, the availability of detailed, visit-level data within a high-volume survivorship program provides a unique perspective into the contemporary role of physical examination within breast cancer follow-up.

## Conclusion

Routine provider-performed breast and chest wall examinations contribute minimally to recurrence detection in modern breast cancer survivorship care. However, survivorship visits remain clinically valuable for symptom assessment, coordination of surveillance imaging, and patient education. These findings support a shift toward survivorship models that emphasize flexible, risk-adapted care delivery strategies that preserve clinical oversight while reducing reliance on low-yield examination-based encounters. Future work may benefit from prospective multi-institutional studies and integration of patient-centered outcomes to inform follow-up frameworks that are evidence-based, sustainable, and responsive to the evolving needs of survivors.

## References

[1] Gradishar WJ, Moran MS, Abraham J, et al. NCCN guidelines® insights: breast cancer, version 5. 2025. *J Natl Compr Canc Netw*. 2025;23(11):426–36. 10.6004/jnccn.2025.0053. 41213254 10.6004/jnccn.2025.0053

[2] Senkus E, Kyriakides S, Penault-Llorca F, et al. Primary breast cancer: ESMO clinical practice guidelines for diagnosis, treatment and follow-up. *Ann Oncol*. 2013;24(Suppl 6):vi7–23. 10.1093/annonc/mdt284. 23970019 10.1093/annonc/mdt284

[3] Runowicz CD, Leach CR, Henry NL, et al. American cancer society/american society of clinical oncology breast cancer survivorship care guideline. *J Clin Oncol*. 2016;34(6):611–35. 10.1200/JCO.2015.64.3809. 26644543 10.1200/JCO.2015.64.3809

[4] Montgomery DA, Krupa K, Cooke TG. Follow-up in breast cancer: does routine clinical examination improve outcome? A systematic review of the literature. *Br J Cancer*. 2007;97(12):1632–41. 10.1038/sj.bjc.6604065. 18000508 10.1038/sj.bjc.6604065 PMC2360278

[5] Ankersmid JW, Drossaert CHC, van Riet YEA, Strobbe LJA, Siesling S, Santeon VBHC. Breast cancer group. Needs and preferences of breast cancer survivors regarding outcome-based shared decision-making about personalised post-treatment surveillance. *J Cancer Surviv*. 2023;17(5):1471–9. 10.1007/s11764-022-01178-z. 35122224 10.1007/s11764-022-01178-z PMC10442247

[6] Beltran-Bless AA, Alshamsan B, Alzahrani MJ, et al. Regularly scheduled physical examinations and the detection of breast cancer recurrences. *Breast*. 2023;69:274–80. 10.1016/j.breast.2023.03.004. 36922304 10.1016/j.breast.2023.03.004 PMC10034490

[7] Beltran-Bless AA, de Lima MI, Clemons L, et al. Do regularly scheduled visits result in earlier detection of curable breast cancer recurrences? *Breast Cancer Res Treat*. 2025;214(1):59–68. 10.1007/s10549-025-07793-5. 40764879 10.1007/s10549-025-07793-5

[8] Godding LTH, Dekker-Klaassen A, Volders JH, et al. The added value of physical examination for breast cancer recurrence detection in women: a systematic review. *Clin Breast Cancer*. 2025;25(4):e383-e393.e2. 10.1016/j.clbc.2024.12.014. 39909790 10.1016/j.clbc.2024.12.014

[9] American Cancer Society: Breast cancer facts & figures 2024-2025. Atlanta: American Cancer Society, Inc. 2024. https://www.cancer.org/content/dam/cancer-org/research/cancer-facts-and-statistics/breast-cancer-facts-and-figures/2024/breast-cancer-facts-and-figures-2024.pdf.

[10] Siegel RL, Giaquinto AN, Jemal A. Cancer statistics, 2024. *CA Cancer J Clin*. 2024;74(1):12–49. 10.3322/caac.21820. 38230766 10.3322/caac.21820

[11] Shih YT, Kim B, Halpern MT. State of physician and pharmacist oncology workforce in the United States in 2019. *JCO Oncol Pract*. 2021;17(1):e1–10. 10.1200/OP.20.00600. 33270520 10.1200/OP.20.00600 PMC8189614

[12] 2022 Snapshot: state of the oncology workforce in America. *JCO Oncol Pract*. 2022;18(5):396. 10.1200/OP.22.00168. 10.1200/OP.22.00168 35544658

[13] Montgomery DA, Krupa K, Jack WJ, et al. Changing pattern of the detection of locoregional relapse in breast cancer: the Edinburgh experience. *Br J Cancer*. 2007;96(12):1802–7. 10.1038/sj.bjc.6603815. 17533401 10.1038/sj.bjc.6603815 PMC2359955

[14] Montgomery DA, Krupa K, Cooke TG. Alternative methods of follow up in breast cancer: a systematic review of the literature. *Br J Cancer*. 2007;96(11):1625–32. 10.1038/sj.bjc.6603771. 17486134 10.1038/sj.bjc.6603771 PMC2359932

[15] van Maaren MC, Strobbe LJA, Smidt ML, Moossdorff M, Poortmans PMP, Siesling S. Ten-year conditional recurrence risks and overall and relative survival for breast cancer patients in the Netherlands: Taking account of event-free years. *Eur J Cancer*. 2018;102:82–94. 10.1016/j.ejca.2018.07.124. 30144661 10.1016/j.ejca.2018.07.124

[16] Holleczek B, Stegmaier C, Radosa JC, Solomayer EF, Brenner H. Risk of loco-regional recurrence and distant metastases of patients with invasive breast cancer up to ten years after diagnosis - results from a registry-based study from Germany. *BMC Cancer*. 2019;19(1):520. 10.1186/s12885-019-5710-5. 31146706 10.1186/s12885-019-5710-5 PMC6543576

[17] Geurts YM, Witteveen A, Bretveld R, et al. Patterns and predictors of first and subsequent recurrence in women with early breast cancer. *Breast Cancer Res Treat*. 2017;165(3):709–20. 10.1007/s10549-017-4340-3. 28677011 10.1007/s10549-017-4340-3 PMC5602040

[18] Liu R, Sundaresan T, Reed ME, Trosman JR, Weldon CB, Kolevska T. Telehealth in oncology during the COVID-19 Outbreak: bringing the house call back virtually. *JCO Oncol Pract*. 2020;16(6):289–93. 10.1200/OP.20.00199. 32364826 10.1200/OP.20.00199

[19] Alfano CM, Jefford M, Maher J, Birken SA, Mayer DK. Building personalized cancer follow-up care pathways in the United States: lessons learned from implementation in England, Northern Ireland, and Australia. *Am Soc Clin Oncol Educ Book*. 2019;39:625–39. 10.1200/EDBK_238267. 31099658 10.1200/EDBK_238267

[20] Saltbæk L, Bidstrup PE, Karlsen RV, et al. Nurse-led individualized follow-up versus regular physician-led visits after early breast cancer (MyHealth): a phase III randomized. *Controlled Trial J Clin Oncol*. 2024;42(17):2038–49. 10.1200/JCO.23.01447. 38498781 10.1200/JCO.23.01447

[21] Graetz I, Hu X, Kocak M, et al. Remote monitoring app for endocrine therapy adherence among patients with early-stage breast cancer: a randomized clinical trial. *JAMA Netw Open*. 2024;7(6):e2417873. 10.1001/jamanetworkopen.2024.17873. 38935379 10.1001/jamanetworkopen.2024.17873 PMC11211959

[22] Ankersmid JW, Siesling S, Strobbe LJA, et al. Supporting shared decision-making about surveillance after breast cancer with personalized recurrence risk calculations: development of a patient decision aid using the international patient decision aids standards development process in combination with a mixed methods design. *JMIR Cancer*. 2022;8(4):e38088. 10.2196/38088. 36374536 10.2196/38088 PMC9706380

[23] Neeman E, Lyon L, Sun H, et al. Future of teleoncology: trends and disparities in telehealth and secure message utilization in the COVID-19 era. *JCO Clin Cancer Inform*. 2022;6:e2100160. 10.1200/CCI.21.00160. 35467963 10.1200/CCI.21.00160 PMC9067360

